# Supplementary material for: Benchmark of biomarker identification and prognostic modeling methods on diverse censored data
Source: PLoS One. 2026 Jun 16;21(6):e0351429. doi: 10.1371/journal.pone.0351429 (PMC13271465; doi:10.1371/journal.pone.0351429)
Supplement: S2 Table — Table rows are partitioned by method and metric. Columns are partitioned data characteristics (α,s,γ). Higher values of CI and F1-score, and lower values of Brier score, RMSE and FDR and computation time indicate better performance. CI: Concordance Index, RMSE: Root Mean Squared Error, FDR: False Discovery Rate. (PDF) [file pone.0351429.s002.pdf]

		$\alpha = 0$									$\alpha = 0.5$								
		$s = 0.02$			$s = 0.05$			$s = 0.1$			$s = 0.02$			$s = 0.05$			$s = 0.1$		
		$\gamma = 0.5$	$\gamma = 1$	$\gamma = 2$	$\gamma = 0.5$	$\gamma = 1$	$\gamma = 2$	$\gamma = 0.5$	$\gamma = 1$	$\gamma = 2$	$\gamma = 0.5$	$\gamma = 1$	$\gamma = 2$	$\gamma = 0.5$	$\gamma = 1$	$\gamma = 2$	$\gamma = 0.5$	$\gamma = 1$	$\gamma = 2$
FDR	LASSO	0.75(0.12)	0.73(0.1)	0.71(0.14)	0.75(0.1)	0.74(0.09)	0.74(0.08)	0.71(0.11)	0.69(0.11)	0.7(0.11)	0.78(0.13)	0.76(0.07)	0.73(0.11)	0.76(0.11)	0.74(0.11)	0.73(0.07)	0.72(0.11)	0.71(0.1)	0.71(0.1)
	ALASSO	0.68(0.07)	0.7(0.07)	0.74(0.04)	0.57(0.08)	0.54(0.09)	0.53(0.09)	0.52(0.11)	0.5(0.1)	0.5(0.1)	0.72(0.1)	0.69(0.06)	0.72(0.04)	0.61(0.12)	0.55(0.09)	0.52(0.09)	0.55(0.14)	0.5(0.12)	0.5(0.13)
	ENET	0.78(0.11)	0.78(0.08)	0.76(0.07)	0.79(0.1)	0.78(0.09)	0.77(0.09)	0.76(0.13)	0.79(0.12)	0.79(0.12)	0.82(0.12)	0.79(0.08)	0.78(0.06)	0.8(0.11)	0.79(0.11)	0.79(0.08)	0.79(0.12)	0.78(0.1)	0.78(0.1)
	CB	0.48(0.12)	0.26(0.14)	0.19(0.13)	0.53(0.1)	0.48(0.11)	0.46(0.1)	0.55(0.1)	0.54(0.11)	0.53(0.1)	0.56(0.15)	0.29(0.17)	0.2(0.17)	0.55(0.15)	0.48(0.14)	0.42(0.14)	0.55(0.14)	0.5(0.14)	0.5(0.14)
	RSF	0.97(0.01)	0.97(0.01)	0.97(0.01)	0.94(0.01)	0.94(0.01)	0.94(0.01)	0.89(0.02)	0.89(0.02)	0.89(0.02)	0.97(0.01)	0.96(0.01)	0.96(0.01)	0.93(0.02)	0.93(0.02)	0.93(0.02)	0.89(0.03)	0.89(0.02)	0.88(0.02)
	sRSF	0.69(0.08)	0.65(0.09)	0.63(0.09)	0.6(0.09)	0.57(0.1)	0.56(0.09)	0.53(0.1)	0.52(0.09)	0.51(0.1)	0.67(0.29)	0.58(0.44)	0.56(0.42)	0.56(0.29)	0.52(0.31)	0.51(0.28)	0.58(0.23)	0.55(0.23)	0.55(0.26)
	BH	**0(0)**	**0(0)**	**0(0)**	**0(0)**	**0(0)**	**0(0)**	**0(0)**	**0(0)**	**0(0)**	**0(0)**	**0(0)**	**0(0)**	**0(0)**	**0(0)**	**0(0)**	**0(0)**	**0(0)**	**0(0)**
	QV	**0(0)**	**0(0)**	**0(0)**	**0(0)**	**0(0)**	**0(0)**	**0(0)**	**0(0)**	**0(0)**	**0(0)**	**0(0)**	**0(0)**	**0(0)**	**0(0)**	**0(0)**	**0(0)**	**0(0)**	**0(0)**
	CARS (MED)	0.92(0.03)	0.9(0.03)	0.88(0.03)	0.86(0.03)	0.84(0.04)	0.83(0.04)	0.8(0.04)	0.79(0.04)	0.78(0.04)	0.93(0.02)	0.9(0.03)	0.88(0.03)	0.87(0.03)	0.84(0.04)	0.83(0.03)	0.81(0.04)	0.79(0.04)	0.78(0.04)
	CARS (MSR)	0.84(0.07)	0.76(0.1)	0.72(0.12)	0.77(0.08)	0.73(0.09)	0.71(0.11)	0.72(0.08)	0.69(0.08)	0.69(0.09)	0.88(0.06)	0.79(0.09)	0.71(0.14)	0.81(0.08)	0.73(0.1)	0.7(0.09)	0.75(0.09)	0.71(0.09)	0.69(0.09)
F1-score	LASSO	0.39(0.14)	0.42(0.13)	0.45(0.17)	0.35(0.09)	0.4(0.09)	0.4(0.08)	**0.33(0.07)**	**0.34(0.06)**	**0.35(0.06)**	0.33(0.14)	0.38(0.09)	0.42(0.13)	0.32(0.09)	0.38(0.11)	0.4(0.08)	**0.31(0.07)**	**0.33(0.06)**	**0.34(0.06)**
	ALASSO	0.47(0.08)	0.47(0.08)	0.41(0.05)	**0.45(0.09)**	**0.51(0.09)**	**0.52(0.08)**	0.3(0.08)	0.31(0.09)	0.32(0.08)	0.39(0.11)	0.47(0.07)	0.44(0.05)	**0.4(0.09)**	**0.49(0.08)**	**0.53(0.07)**	0.28(0.09)	0.32(0.09)	0.32(0.1)
	ENET	0.35(0.14)	0.36(0.11)	0.38(0.09)	0.33(0.11)	0.35(0.11)	0.36(0.1)	0.31(0.05)	0.31(0.05)	0.31(0.05)	0.29(0.14)	0.34(0.1)	0.36(0.08)	0.3(0.11)	0.33(0.13)	0.34(0.1)	0.29(0.05)	0.31(0.06)	0.32(0.05)
	CB	**0.61(0.11)**	**0.8(0.11)**	**0.85(0.1)**	0.39(0.08)	0.43(0.08)	0.44(0.07)	0.25(0.05)	0.26(0.05)	0.26(0.05)	**0.47(0.14)**	**0.68(0.13)**	**0.75(0.11)**	0.31(0.09)	0.35(0.09)	0.37(0.1)	0.19(0.06)	0.21(0.06)	0.21(0.05)
	RSF	0.05(0.02)	0.06(0.02)	0.06(0.02)	0.1(0.02)	0.1(0.02)	0.1(0.02)	0.17(0.03)	0.17(0.03)	0.17(0.03)	0.06(0.02)	0.07(0.02)	0.07(0.03)	0.11(0.03)	0.12(0.03)	0.12(0.03)	0.17(0.04)	0.17(0.04)	0.17(0.03)
	sRSF	0.41(0.1)	0.46(0.09)	0.48(0.09)	0.36(0.08)	0.39(0.08)	0.38(0.08)	0.27(0.06)	0.28(0.06)	0.26(0.05)	0.29(0.15)	0.38(0.22)	0.4(0.2)	0.23(0.09)	0.25(0.1)	0.25(0.1)	0.16(0.14)	0.17(0.12)	0.16(0.13)
	BH	0.18(0.2)	0.31(0.22)	0.33(0.17)	0(0.04)	0(0.04)	0(0.04)	0(0)	0(0)	0(0)	0(0)	0(0)	0(0.1)	0(0)	0(0)	0(0)	0(0)	0(0)	0(0)
	QV	0.18(0.22)	0.32(0.22)	0.33(0.18)	0(0.04)	0(0.04)	0(0.04)	0(0)	0(0)	0(0)	0(0)	0(0.04)	0(0.04)	0(0)	0(0)	0(0)	0(0)	0(0)	0(0)
	CARS (MED)	0.15(0.04)	0.18(0.05)	0.2(0.06)	0.22(0.04)	0.24(0.05)	0.25(0.05)	0.24(0.04)	0.26(0.04)	0.27(0.04)	0.12(0.04)	0.17(0.05)	0.2(0.05)	0.19(0.05)	0.23(0.05)	0.25(0.04)	0.23(0.03)	0.26(0.04)	0.27(0.05)
	CARS (MSR)	0.23(0.08)	0.32(0.1)	0.37(0.1)	0.23(0.07)	0.27(0.08)	0.28(0.07)	0.19(0.07)	0.21(0.06)	0.21(0.07)	0.17(0.07)	0.29(0.1)	0.38(0.12)	0.19(0.08)	0.25(0.08)	0.29(0.08)	0.18(0.07)	0.2(0.07)	0.22(0.07)
CI	LASSO	**0.75(0.05)**	0.88(0.03)	**0.95(0.01)**	0.66(0.06)	**0.72(0.06)**	**0.77(0.08)**	0.61(0.06)	0.63(0.06)	0.66(0.08)	0.66(0.06)	**0.83(0.04)**	**0.92(0.02)**	**0.63(0.06)**	**0.69(0.05)**	**0.74(0.05)**	0.6(0.06)	0.62(0.06)	0.64(0.07)
	ALASSO	**0.75(0.06)**	**0.89(0.03)**	**0.95(0.01)**	0.65(0.06)	0.7(0.06)	0.73(0.07)	0.6(0.05)	0.61(0.06)	0.63(0.07)	**0.67(0.06)**	**0.83(0.05)**	**0.92(0.02)**	**0.63(0.05)**	0.68(0.05)	0.71(0.06)	0.59(0.06)	0.61(0.05)	0.62(0.06)
	ENET	0.74(0.06)	0.86(0.03)	0.92(0.02)	**0.67(0.05)**	0.71(0.06)	0.75(0.07)	**0.63(0.06)**	**0.66(0.06)**	**0.68(0.06)**	0.65(0.06)	0.8(0.05)	0.88(0.03)	**0.63(0.05)**	**0.69(0.05)**	0.72(0.05)	**0.62(0.06)**	**0.64(0.05)**	**0.66(0.05)**
	CB	0.71(0.06)	0.78(0.05)	0.81(0.04)	0.63(0.05)	0.64(0.05)	0.67(0.06)	0.59(0.04)	0.6(0.05)	0.61(0.06)	0.64(0.06)	0.71(0.05)	0.74(0.05)	0.59(0.05)	0.61(0.05)	0.62(0.06)	0.56(0.05)	0.57(0.06)	0.58(0.06)
	RSF	0.52(0.06)	0.52(0.05)	0.53(0.05)	0.52(0.05)	0.52(0.05)	0.52(0.05)	0.51(0.05)	0.52(0.05)	0.51(0.06)	0.51(0.06)	0.52(0.05)	0.52(0.05)	0.51(0.05)	0.51(0.04)	0.51(0.05)	0.51(0.05)	0.5(0.05)	0.51(0.06)
	sRSF	0.57(0.05)	0.59(0.05)	0.59(0.05)	0.55(0.05)	0.56(0.05)	0.56(0.05)	0.55(0.06)	0.55(0.05)	0.55(0.06)	0.54(0.06)	0.57(0.08)	0.58(0.08)	0.53(0.06)	0.54(0.06)	0.54(0.06)	0.52(0.06)	0.52(0.05)	0.52(0.06)
	BH	0.58(0.08)	0.63(0.08)	0.65(0.07)	0.5(0.05)	0.5(0.05)	0.5(0.04)	0.5(0)	0.5(0)	0.5(0)	0.5(0)	0.5(0)	0.5(0)	0.5(0)	0.5(0)	0.5(0)	0.5(0)	0.5(0)	0.5(0)
	QV	0.59(0.09)	0.64(0.08)	0.65(0.07)	0.5(0.05)	0.5(0.05)	0.5(0.04)	0.5(0)	0.5(0)	0.5(0)	0.5(0)	0.5(0.01)	0.5(0.02)	0.5(0)	0.5(0)	0.5(0)	0.5(0)	0.5(0)	0.5(0)
	CARS (MED)	0.58(0.07)	0.66(0.08)	0.7(0.09)	0.56(0.06)	0.58(0.07)	0.59(0.07)	0.54(0.05)	0.55(0.05)	0.55(0.08)	0.55(0.08)	0.63(0.09)	0.7(0.1)	0.55(0.06)	0.58(0.07)	0.59(0.07)	0.54(0.06)	0.56(0.05)	0.55(0.07)
	CARS (MSR)	0.63(0.06)	0.69(0.07)	0.72(0.06)	0.59(0.05)	0.61(0.05)	0.62(0.05)	0.57(0.05)	0.57(0.05)	0.58(0.07)	0.58(0.06)	0.66(0.08)	0.71(0.08)	0.58(0.06)	0.6(0.06)	0.62(0.05)	0.56(0.05)	0.57(0.05)	0.58(0.05)
Brier score	LASSO	0.23(0.07)	**0.11(0.05)**	**0.08(0.03)**	0.35(0.14)	0.32(0.16)	0.36(0.19)	0.38(0.14)	0.39(0.16)	0.41(0.28)	0.29(0.09)	0.17(0.06)	**0.08(0.04)**	0.35(0.11)	0.32(0.12)	0.33(0.16)	0.38(0.13)	0.39(0.14)	0.4(0.15)
	ALASSO	0.21(0.06)	**0.11(0.04)**	**0.08(0.03)**	0.27(0.05)	**0.23(0.06)**	**0.22(0.04)**	0.29(0.05)	0.28(0.05)	0.25(0.04)	0.27(0.08)	**0.15(0.05)**	**0.08(0.04)**	0.29(0.05)	**0.25(0.06)**	**0.23(0.05)**	0.29(0.05)	0.29(0.06)	0.27(0.04)
	ENET	0.25(0.08)	0.13(0.06)	0.1(0.04)	0.43(0.16)	0.37(0.18)	0.45(0.17)	0.43(0.16)	0.46(0.14)	0.57(0.21)	0.3(0.12)	0.19(0.08)	0.09(0.04)	0.41(0.17)	0.41(0.2)	0.46(0.16)	0.46(0.16)	0.44(0.12)	0.47(0.12)
	CB	**0.19(0.05)**	**0.11(0.05)**	0.09(0.04)	0.27(0.05)	0.24(0.05)	0.23(0.04)	0.29(0.04)	0.28(0.04)	0.26(0.04)	**0.24(0.05)**	0.17(0.04)	0.15(0.04)	0.27(0.04)	**0.25(0.04)**	0.25(0.04)	0.29(0.04)	0.28(0.04)	0.27(0.04)
	RSF	0.26(0.02)	0.26(0.02)	0.26(0.02)	0.26(0.02)	0.26(0.02)	0.26(0.02)	0.26(0.02)	0.26(0.02)	0.26(0.02)	0.28(0.03)	0.28(0.03)	0.28(0.03)	0.28(0.03)	0.28(0.03)	0.28(0.03)	0.28(0.03)	0.28(0.03)	0.28(0.03)
	sRSF	0.25(0.02)	0.24(0.02)	0.24(0.02)	**0.25(0.02)**	0.25(0.02)	0.25(0.02)	0.26(0.02)	0.26(0.02)	0.26(0.02)	0.26(0.03)	0.25(0.03)	0.25(0.03)	0.26(0.03)	0.26(0.03)	0.26(0.03)	0.27(0.02)	0.27(0.03)	0.27(0.03)
	BH	0.25(0.02)	0.23(0.03)	0.22(0.03)	**0.25(0.01)**	0.25(0.01)	0.25(0.01)	**0.25(0.01)**	**0.25(0.01)**	**0.24(0.02)**	0.25(0.01)	0.25(0)	0.25(0)	**0.25(0)**	**0.25(0)**	0.25(0)	**0.25(0)**	**0.25(0)**	**0.25(0)**
	QV	0.25(0.02)	0.23(0.03)	0.22(0.03)	**0.25(0.01)**	0.25(0.01)	0.25(0.01)	**0.25(0.01)**	**0.25(0.01)**	**0.24(0.02)**	0.25(0.01)	0.25(0.01)	0.25(0)	**0.25(0)**	**0.25(0)**	0.25(0)	**0.25(0.01)**	**0.25(0)**	**0.25(0)**
	CARS (MED)	0.39(0.11)	0.31(0.11)	0.25(0.1)	0.41(0.08)	0.38(0.09)	0.37(0.11)	0.42(0.09)	0.41(0.09)	0.4(0.14)	0.41(0.1)	0.34(0.12)	0.26(0.1)	0.41(0.09)	0.38(0.1)	0.36(0.12)	0.43(0.1)	0.41(0.1)	0.4(0.11)
	CARS (MSR)	0.26(0.05)	0.22(0.05)	0.19(0.04)	0.28(0.05)	0.27(0.05)	0.25(0.03)	0.29(0.06)	0.29(0.05)	0.27(0.04)	0.28(0.05)	0.23(0.05)	0.2(0.06)	0.29(0.05)	0.27(0.05)	0.26(0.04)	0.3(0.05)	0.29(0.05)	0.28(0.04)
RMSE	LASSO	2.02(0.3)	2.47(0.57)	**4.5(1.42)**	3.61(0.56)	6.62(1.07)	18.36(7.69)	5.33(0.7)	11.11(2.86)	36.64(20.98)	1.93(0.31)	2.18(0.45)	3.2(0.89)	2.82(0.31)	4.78(0.85)	10.46(1.87)	3.9(0.59)	7.3(0.99)	19.95(9.36)
	ALASSO	**1.94(0.29)**	2.36(0.4)	4.53(1.2)	**3.47(0.41)**	**6.12(0.83)**	20.79(5.02)	5.22(0.66)	12.1(2.12)	43.21(8.64)	1.88(0.27)	**2.02(0.36)**	**3.19(0.6)**	**2.76(0.33)**	**4.48(0.57)**	10.33(1.8)	3.85(0.38)	7.25(0.83)	23.72(5.35)
	ENET	2.11(0.34)	2.63(0.63)	4.97(1.3)	3.86(0.89)	6.87(1.03)	16.1(7.07)	5.43(1.01)	10.76(1.88)	23.53(13.92)	1.97(0.27)	2.33(0.52)	3.33(0.88)	2.99(0.67)	5.1(1.1)	10.41(1.63)	4.12(1.2)	**7.14(1.15)**	15.34(4.43)
	CB	1.96(0.31)	**2.34(0.63)**	5.14(1.35)	3.62(0.45)	6.66(0.86)	22.19(4.64)	5.31(0.56)	12.57(2.2)	43.58(9.16)	**1.86(0.28)**	2.43(0.37)	4.05(0.74)	2.84(0.3)	4.95(0.56)	11.74(2.13)	3.94(0.4)	7.46(0.75)	24.36(5.71)
	RSF	2.63(0.25)	4.77(0.49)	9.25(0.88)	3.91(0.39)	7.43(0.73)	14.73(1.55)	5.37(0.56)	10.45(1.14)	20.81(2.25)	2.24(0.24)	3.73(0.34)	7.04(0.68)	3.14(0.31)	5.72(0.55)	11.17(1.18)	4.18(0.45)	7.97(0.87)	15.72(1.62)
	sRSF	2.56(0.24)	4.52(0.5)	8.76(0.89)	3.82(0.37)	7.26(0.67)	**14.27(1.44)**	5.31(0.56)	10.28(1.1)	**20.48(2.37)**	2.11(0.23)	3.42(0.41)	6.36(0.68)	2.94(0.32)	5.36(0.54)	10.47(1.1)	3.97(0.45)	7.62(0.88)	15.03(1.63)
	BH	2.49(0.25)	4.28(0.48)	8.5(1.12)	3.79(0.4)	7.22(0.69)	15.87(13.78)	**5.2(0.52)**	**10.17(1.2)**	56.61(11.92)	2.04(0.2)	3.4(0.29)	6.41(0.57)	2.81(0.27)	5.17(0.47)	**10.12(1.03)**	**3.76(0.37)**	**7.14(0.72)**	**14.24(1.81)**
	QV	2.49(0.25)	4.27(0.49)	8.5(1.13)	3.79(0.4)	7.22(0.69)	15.95(13.52)	**5.2(0.52)**	10.19(1.2)	56.6(11.94)	2.05(0.21)	3.42(0.3)	6.43(0.57)	2.82(0.28)	5.18(0.47)	**10.12(1)**	3.79(0.45)	**7.14(0.69)**	**14.28(2.04)**
	CARS (MED)	3.28(0.9)	4.74(1.29)	9.71(2.61)	5(1.03)	9.19(2.71)	26.38(6.47)	7.05(1.8)	16.75(4.89)	44.2(11.92)	2.85(0.82)	3.75(1.03)	5.81(1.5)	3.75(0.91)	6.35(1.37)	14.78(3.94)	5(1.14)	9.62(2.39)	27.25(6.86)
	CARS (MSR)	2.49(0.35)	3.91(0.67)	8.12(1.22)	3.92(0.48)	7.3(0.83)	24.9(5.05)	5.5(0.75)	13.27(2.41)	46.69(9.4)	2.11(0.29)	3.04(0.49)	5.11(0.83)	2.96(0.34)	5.21(0.54)	12.31(2.47)	4.06(0.45)	7.65(0.77)	26.16(6.08)
Computation time	LASSO	36.43(3.85)	39.31(4.14)	41.71(4.79)	36.24(4.12)	37.36(4.35)	37.8(4.61)	36.4(3.54)	37.56(3.93)	38.18(4.13)	35.78(6.02)	41.44(7.45)	48.77(8.46)	35.97(5.23)	38.01(6)	38.71(6.14)	36.6(6.38)	38.07(7.97)	37.58(7.87)
	ALASSO	1.04(0.35)	1.08(0.24)	1.04(0.31)	1(0.42)	1(0.57)	1.03(0.49)	1.06(0.51)	1.06(0.57)	1.07(0.49)	1.72(0.38)	1.6(0.41)	1.53(0.47)	1.89(0.5)	1.86(0.55)	1.87(0.62)	2.03(0.49)	2.07(0.59)	2.06(0.54)
	ENET	18.98(1.75)	18.53(1.54)	18.36(1.88)	19.6(1.87)	19.74(1.85)	20(2.03)	20.46(2.67)	21.16(2.83)	21.73(3)	21.04(3.07)	21.77(2.87)	22.47(3.2)	22.27(2.68)	23.02(2.6)	23.46(2.79)	23.55(3.79)	23.69(4.09)	23.74(5.02)
	CB	4.92(0.27)	5(0.09)	5.06(0.08)	4.95(0.11)	5.05(0.1)	5.09(0.08)	5.01(0.09)	5.08(0.09)	5.1(0.09)	4.81(0.15)	4.95(0.11)	5.05(0.09)	4.9(0.13)	5.03(0.1)	5.09(0.08)	4.97(0.12)	5.05(0.12)	5.1(0.14)
	RSF	365.22(18.93)	377.24(18.68)	383.77(15.12)	373.25(16.48)	387.79(13.15)	392.33(14.89)	382.58(15.19)	391.05(14.48)	393.84(13.25)	200.43(28.26)	205.34(28.27)	210.76(29.17)	208.93(28.55)	215.64(35.35)	219.3(35.22)	209.91(33.61)	214.56(38.76)	213.94(47.54)
	sRSF	56.97(9.94)	59.1(9.51)	59.3(9.21)	62.36(9.13)	64.58(9.95)	60.89(9.31)	66.02(11.2)	66.21(11.48)	55.47(10.18)	26.46(34.86)	27.4(40.21)	28.27(39.85)	25.26(26.81)	26.83(23.64)	26.36(29.09)	32.87(49.15)	30.62(43.27)	25.77(37.62)
	BH	4.75(0.55)	4.76(0.35)	4.85(0.29)	4.74(0.25)	4.88(0.2)	4.84(0.24)	4.79(0.26)	4.85(0.21)	4.83(0.23)	4.77(0.19)	4.75(0.2)	4.94(0.17)	4.79(0.19)	4.86(0.21)	4.91(0.19)	4.77(0.37)	4.87(0.89)	4.82(2.32)
	QV	4.75(0.47)	4.76(0.34)	4.83(0.28)	4.72(0.24)	4.86(0.24)	4.81(0.24)	4.8(0.24)	4.83(0.19)	4.83(0.23)	4.75(0.17)	4.74(0.16)	4.92(0.19)	4.77(0.17)	4.84(0.2)	4.9(0.18)	4.75(0.53)	4.88(1.23)	4.82(1.43)
	CARS (MED)	**0.26(0.38)**	**0.25(0.02)**	**0.25(0.02)**	**0.24(0.02)**	**0.25(0.02)**	**0.25(0.02)**	**0.25(0.02)**	**0.25(0.02)**	**0.25(0.02)**	**0.26(0.03)**	**0.25(0.02)**	**0.25(0.02)**	**0.25(0.02)**	**0.25(0.03)**	**0.26(0.03)**	**0.25(0.04)**	**0.25(0.03)**	**0.24(0.03)**
	CARS (MSR)	3.7(0.14)	3.7(0.13)	3.72(0.11)	3.71(0.11)	3.71(0.11)	3.71(0.13)	3.71(0.12)	3.73(0.11)	3.73(0.12)	3.74(0.1)	3.74(0.11)	3.75(0.11)	3.75(0.1)	3.75(0.09)	3.74(0.1)	3.72(0.33)	3.72(0.12)	3.71(1.01)
