# Supplementary material for: Benchmark of biomarker identification and prognostic modeling methods on diverse censored data
Source: PLoS One. 2026 Jun 16;21(6):e0351429. doi: 10.1371/journal.pone.0351429 (PMC13271465; doi:10.1371/journal.pone.0351429)
Supplement: S3 Table — The 30 mRNA features likely associated with differences in survival time listen in Table 2 are listed over the rows, and the examined methods are provided in the columns. Each cell corresponds to how many of the ten outer folds the examined method selected the respective mRNA feature. (PDF) [file pone.0351429.s003.pdf]

Known driver	LASSO	ALASSO	ENET	CB	RSF	sRSF	BHP	QV	CARS (MED)	CARS (MSR)
ATG5	0	0	0	0	0	0	0	0	0	0
DLEU1	9	7	8	10	1	3	9	10	0	0
FGF14	0	0	0	0	1	0	0	0	0	0
FGF22	0	0	0	0	1	0	0	0	0	0
FGF5	0	0	0	0	0	0	0	0	0	0
FGFRL1	0	0	0	0	0	0	0	0	0	0
FOXA3	0	0	0	0	0	0	0	0	0	0
FOXF1	0	0	0	0	2	0	0	0	0	0
FOXF2	0	0	0	0	0	0	0	0	0	0
FOXG1	0	0	0	0	1	0	0	0	0	0
FOXI1	1	0	1	0	4	0	0	0	0	0
FOXK2	0	0	0	0	3	0	0	0	0	0
FOXL2	0	0	0	0	0	0	0	0	0	0
FOXN4	0	0	0	0	2	0	0	0	0	0
FOXP3	0	0	0	0	8	1	0	0	0	0
FOXR1	0	0	0	0	0	0	0	0	0	0
IGF1R	0	0	0	0	1	0	0	0	0	0
IGF2BP1	0	0	0	0	0	0	0	0	0	0
MMP19	2	0	1	0	3	0	0	0	0	0
MMP20	0	0	0	0	4	0	0	0	0	0
MMP27	0	0	0	0	10	5	0	0	3	0
MMP8	0	0	0	0	2	2	0	0	1	0
PLA2G3	0	0	0	0	0	0	0	0	0	0
PLA2G4A	0	0	0	0	2	0	0	0	0	0
PLA2G4D	0	0	0	0	0	0	0	0	0	0
PSMD13	0	0	0	0	0	0	0	0	0	0
PSMD4	0	0	0	0	1	0	0	0	0	0
SQLE	0	0	0	0	2	1	0	0	0	0
TOP1	0	0	0	0	0	0	0	0	0	0
TOP3B	2	1	1	3	10	10	10	10	0	0
