# Supplementary figures and images for: Benchmark of biomarker identification and prognostic modeling methods on diverse censored data
Source: PLoS One. 2026 Jun 16;21(6):e0351429. doi: 10.1371/journal.pone.0351429 (PMC13271465; doi:10.1371/journal.pone.0351429)

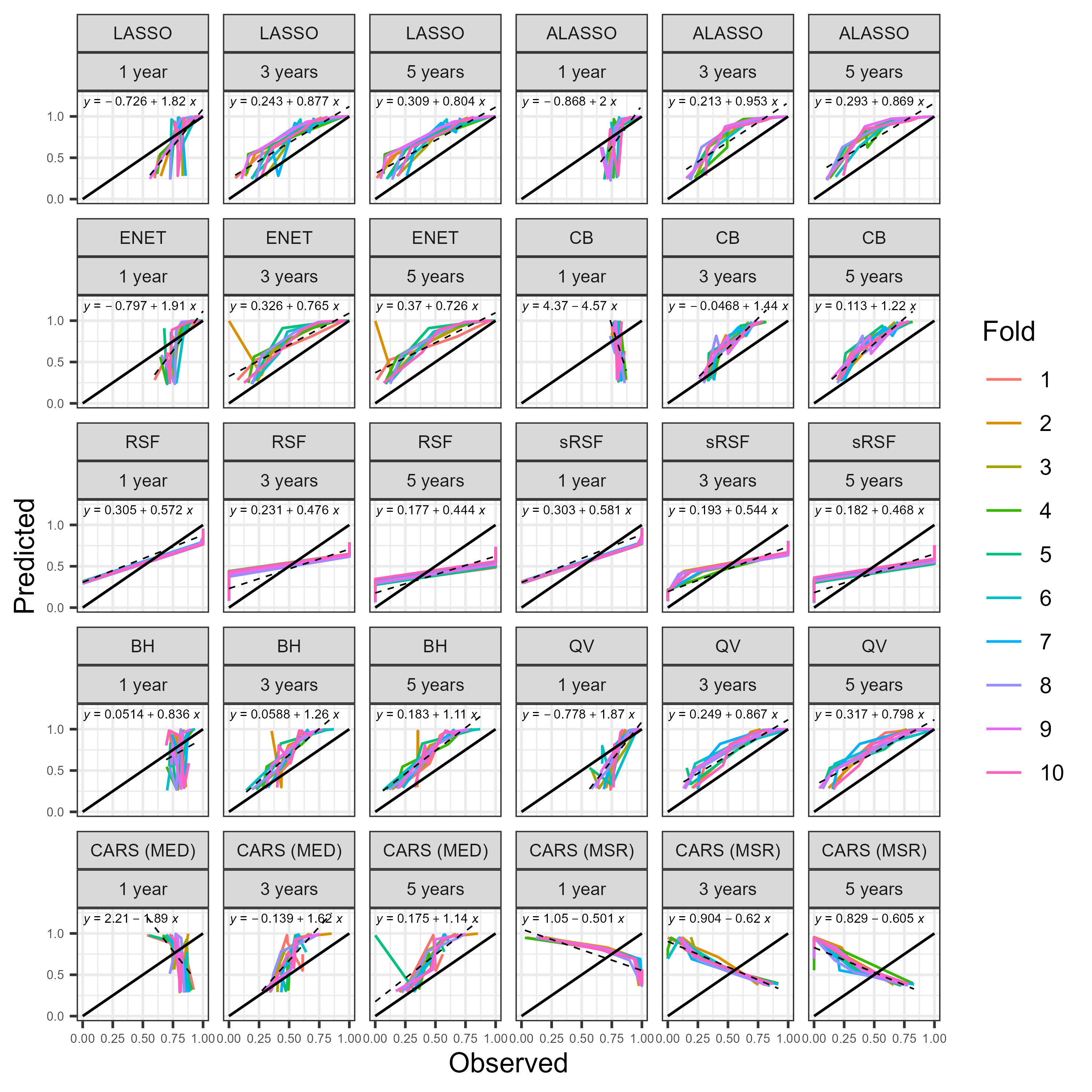

Supplement: S4 Fig — The calibration curves of predicted-on-observed survival probabilities are faceted by method and clinical horizon. The curves for each of the 10 folds are colored and overlayed within each subplot. Well-calibrated methods have curves near the y = x line, shown in solid black. Least squares regression lines are shown per method and horizon as dashed black lines, with their intercepts and slopes provided in each subplot. (JPEG) [file pone.0351429.s004.jpeg]
