# Supplementary material for: Benchmark of biomarker identification and prognostic modeling methods on diverse censored data
Source: PLoS One. 2026 Jun 16;21(6):e0351429. doi: 10.1371/journal.pone.0351429 (PMC13271465; doi:10.1371/journal.pone.0351429)
Supplement: Attachment — Submitted filename: Response to Reviewers.pdf [file pone.0351429.s006.pdf]

Response to the Reviewer 1

We would like to thank you for reading the article and providing valuable suggestions. In the point-by-point response, for your convenience, your comments are italicized followed by our responses. All edits on the paper are in blue.

This manuscript investigates the comparative performance of methods for feature selection and survival prediction in high-dimensional right-censored survival data, using two simulation settings and a TCGA bladder cancer cohort.

Comments:

1. Sparsity levels. The paper states sparsity levels of 2%, 5%, and 10%, but on p.12 (Predictive performances), it refers to 1% and 5%.

Our response: Thank you for noting this discrepancy, it is corrected in the revised manuscript.

2. Suggestion for Figures 7–9. The results are primarily presented with boxplots, which show relative ordering but make it hard for readers to see the size of the differences. Adding a compact summary table with the median CI and median RMSE for each method would make the improvements clearer.

Our response: Thank you for this suggestion. A summary statistics table (S2 Table) of median values and inter-quartile ranges for all metrics is now presented in the supplementary materials with reference in the main text. Since there is a total of 1,080 entries, we present it in the supplementary materials instead of the main document.

Response to the Reviewer 2

We would like to thank you for reading the article and providing valuable suggestions. In the point-by-point response, for your convenience, your comments are italicized followed by our responses. All edits on the paper are in blue.

The study examined several existing analysis methods to compare variable selection and survival prediction on both synthetic and TCGA dataset. The authors then manipulated features in generating synthetic datasets and evaluated the performance of each method using various criteria. Based on their results, the authors recommended ALASSO and CoxBoost as the most effective, among other recommendations. The overall significance and quality of the study can be further improved by addressing the following:
1. Line 271: Is there a particular reason the bladder cancer cohort is selected among other disease types and datasets available in the TCGA?

Our response: We included analysis on the TCGA bladder cancer cohort to demonstrate the application of the methods on a real dataset. Of the TCGA cohorts, the bladder cancer cohort in particular was chosen as it had a similar number of observations to those we generated in our simulation study, and had few missing fields. For conciseness, we decided to only perform analyses on one cohort. We want to emphasize that analysis of this data is not for benchmarking the methods but to illustrate how the methods can be applied to real data.

2. The authors reported DLEU1 deletion was selected by all methods except CARS and MSR. Was this target found or validated previously through experimental methods? Is it logically sound to assume that, with such rigorous methodology, well known drivers with mRNA level differences in various cancers should be discovered, and may serve as positive controls to the methods used in the study? If so, such observations can add credibility to the results and methods of the study.

Our response: Thank you for the suggestion of considering a list of known positive controls for better evaluating feature selection capability in the real data analysis. We have compiled a list of known disease drivers from existing literature and cross-referenced this list with the features selected by each method in this analysis. We want to emphasize that in the absence of the complete list of disease drivers, false discovery rate and F1-Score metrics cannot be determined, and so simpler summary statistics are measured instead, along with a selection stability metric (the Dice

coefficient). We have made corresponding adjustments to the manuscript that outline the exact changes, and supplemented the previous feature selection portion of the real data analysis with the new study design for conciseness. The list of found disease drivers from existing literature is also provided in the manuscript (Table 2).

Response to the Reviewer 3

We would like to thank you for reading the article and providing valuable suggestions. In the point-by-point response, for your convenience, your comments are italicized followed by our responses. All edits on the paper are in blue.

The paper should explicitly document where each data operation happens relative to cross-validation. Any feature screening, pilot fits for adaptive penalties, or hyperparameter tuning must be repeated strictly within training folds for every split. A concise data-flow diagram per fold would remove ambiguity. If nested cross-validation was not used for hyperparameter selection, please switch to it or justify the choice and quantify any optimistic bias.

Our response: Thank you for the suggestions, we have added data flow diagrams to the manuscript for each portion of the study (Figures 2, 3, and 6). In both settings of simulations, we partition the data so that some observations are withheld from all model training and are used exclusively for evaluating out-of-sample predictive performance. For methods that use hyperparameter tuning, 10-fold cross validation is performed on only the training set, and after cross-validation is performed, all observations from the training set are used for model fitting. Instead of performing multiple iterations of this procedure on the same dataset with an outer loop, we instead generate new synthetic datasets. In this way, although we do not necessarily perform nested cross validation, no optimistic bias is introduced in the predictive metrics with our approach. For the real data case, we use nested cross validation. The new data flow diagrams better illustrate the processes used by each component of the study, and we have briefly discussed this issue in the subsection "Real data analysis: Analysis techniques."

Tuning budgets, search spaces, and early-stopping criteria appear uneven across algorithms. Key hyperparameters are not tuned under a common budget or search design across methods (e.g., broad λ paths for penalized Cox contrasted with largely default RSF settings), and there's no nested, evaluation-count–matched inner loop; as a result, observed performance may conflate model quality with unequal search effort.

Our response: Thank you for your feedback on the discrepancy of hyperparameter search efforts between the examined methods. We have since performed an additional simulation study outside

of the benchmark to tune the RSF for a general case, and used the estimated optimal hyperparameters in all parts of the benchmark study. Hyperparameter tuning was not done separately on every dataset due to computational infeasibility in our large-scale simulation study with eighteen different scenarios and 200 replications for each scenario. Brief discussion of this optimistic *a priori* hyperparameter tuning has been appended to the manuscript, along with a statement on how this choice may impact the method's comparative performance. To quantify the performance difference, we performed an additional simulation using RSF with default hyperparameters, our *a priori* tuned hyperparameters, and with hyperparameters tuned for each individual dataset at runtime. The following table shows the out-of-sample concordance index (CI) and computation time of these three assignments of hyperparameters (The R package defaults, our assignments used in the study, and hyperparameters tuned for each individual dataset at runtime respectively). Entries are the mean CI (standard deviation) across the 20 replications, and the mean computation time (standard deviation) for a single replication. The details of this simulation are given at the beginning of the supplementary appendix, under the subtitle "Impact of RSF tuning parameters on performance". The results clearly show very little or no difference in the predictive performance (measured in out-of-sample concordance index) between the three variants, but unsurprisingly extreme differences in computation time when hyperparameters are tuned for each dataset.

Hyperparameters	CI	Computation time (in seconds)
Default	0.52(0.03)	83.24(0)
a priori	0.51(0.03)	100.8(8.49)
Tuned at runtime	0.51(0.03)	1191.47(125.73)

For the TCGA analysis, add time-dependent Brier score (or integrated Brier), and/or calibration assessments at clinically relevant horizons (e.g., 1- and 3-year). Make it explicit that RMSE on log-time is simulation-only since true event times are not observed with censoring in real data.

Our response: Thank you for this suggestion. We have added the Brier score at 365 days and 1,000 days time point to the TCGA analysis, and at other time points relevant to the true underlying distributions of survival times to both settings of simulations. Please see the subsections "Simulation study: Predictive capability metrics: Brier score" and "Real data analysis: Analysis techniques" of the revised manuscript. We have also added the above note for RMSE in the manuscript immedi-

ately following the definition of the metric, as well as an additional note in the description of the real data analysis.

In the absence of ground truth for TCGA, please report selection stability. Bootstrapped selection frequencies, overlap heatmaps, and simple stability-selection summaries will help readers judge robustness. If conclusions about specific genes are highlighted, include their selection frequency and effect direction across folds.

Our response: Thank you for your suggestions for improvements to the TCGA analysis. The analysis has been modified significantly to quantify the examined methods' ability to select features among a list of known disease drivers found within existing literature. Summary statistics from the bootstrapped dataset are now provided in the manuscript that may help readers judge the feature selection accuracies and consistencies of the methods in the real data case. We have also calculated the Dice coefficient on each method's set of selected features over all folds of the TCGA analysis to show selection stability, presented in Table 5. Gene selection frequencies of the 30 known disease drivers are provided in S3 Table.

Because the preliminary screening step materially shapes downstream results, quantify its impact. In simulations, show the rate at which true signals are removed by screening under varying signal-to-noise; in TCGA, present at least one robustness check where embedded methods are run with and without preliminary screening.

Our response: Thank you for your feedback on the preliminary screening step. Following your suggestion we have also performed an additional simulation study where the embedded methods are ran with and without the preliminary screening step to quantify how performance may have been affected by screening. The details of this simulation and the results can be found in our supplementary appendix, under the subtitle "Effect of PFS on embedded methods' performances in the real data analysis."

Regarding the TCGA analysis, we have modified the preliminary screening to use the CARS method as opposed to a multi-colinearity based screening (our past version). The feature selection performance of CARS can be observed in the main benchmark results. We also performed an additional simulation study to quantify the removal rate of true signals by PFS for varying signal-to-noise ratios, which is provided in our supplementary appendix under the subtitle "Retention

of true signals by PFS under varying signal-to-noise ratios".

Ensure the benchmark isn't biased toward L1-only Cox. So at least add Elastic-Net Cox ($\alpha \in$ {0.2, 0.5, 0.8}; tune λ with inner CV). This directly addresses correlated features—a common reality in omics—and materially affects conclusions about "best" pipelines.

Our response: Thank you for the suggestion, the Elastic-Net method has been added to the study. For conciseness, we consider the fixed $\alpha = 0.5$ case with λ tuned by cross-validation.

Results on a single TCGA cohort limit generality. If feasible, add a second cancer type with similar preprocessing to show whether method rankings persist. If not, clearly caveat that BLCA is illustrative and avoid claims of general superiority.

Our response: Thank you for the suggestion, we have placed greater emphasis in the manuscript on how the TCGA analysis is illustrative and not the primary component of the benchmark. The primary component of benchmarking is the analysis of the synthetic data where the truth is known.

Provide fixed seeds, fold assignments, and a repository snapshot (commit hash or DOI) with scripts to regenerate figures and tables. Export per-fold predictions and outcomes so third parties can independently recompute CI, Brier, and calibration. This materially increases the paper's value as a community benchmark.

Our response: Thank you for the suggestion. We have expanded our repository to contain R code for executing all portions of the benchmark study with our fixed seeds/folds as well as reproducing the figures and tables, with a brief user's guide. Please see the updated repository at https://github.com/WesLFletch/censored-data-regression-benchmark/, commit hash 67f6a620839b71b80231b4c954106fc60efdc969.

Minor comments

Standardize "censoring rate" (not "censorship"), correct typos, and ensure consistent naming of methods and packages.

Our response: Thank you, done.

Clarify which q-value implementation is used and specify π_0 estimation; if a nonstandard helper is employed, justify or switch to the canonical qvalue package for clarity.

Our response: Thank you for the inquiry. We used the default π_0 "smoother" estimator (Storey and Tibshirani, 2003, PNAS, 100, 9440–9445) from the `WGCNA` package q-value method implementation. The manuscript has been revised to include this information.

For RSF, compare OOB and held-out metrics to rule out tuning mismatch. If you introduce any heuristic thresholding (e.g., MSR "elbow"), add a brief sensitivity analysis to show conclusions are not fragile to that choice.

Our response: Thank you for the feedback; we have included a brief sensitivity analysis of the heuristic thresholding methods to the main manuscript immediately following their definitions. We have also included an additional simulation study comparing out-of-bag and held-out predictive evaluations of the RSF in the supplementary appendix.

Finally, add a short "methods-at-a-glance" table summarizing each pipeline (what is tuned, what is penalized, what the output is, and typical runtime) to improve usability for non-experts.

Our response: Thank you for the suggestion, we have added a new table to the manuscript that briefly summarizes each method (Table 1).

Response to the Reviewer 4

We would like to thank you for reading the article and providing valuable suggestions. In the point-by-point response, for your convenience, your comments are italicized followed by our responses. All edits on the paper are in blue.

In this study, the authors compared various methods for identifying genomic biomarkers and developing prognostic models for time-to-event (survival) data. Genomic data are typically high-dimensional, correlated, and sparse, which makes feature selection (i.e., identifying informative biomarkers) challenging. Building upon existing methods that address these issues, the authors conducted a large-scale benchmark analysis using TCGA data to evaluate model performance across diverse right-censored survival datasets. By assessing multiple metrics—including Concordance Index, F1-Score, False Discovery Rate, Root Mean Square Error, and computation time—they found that CoxBoost performed well across all metrics, while LASSO and Adaptive LASSO achieved strong performance in terms of Concordance Index and F1-Score. The study is well-designed, and the logical structure is sound. However, I have several concerns:

1. The synthetic dataset includes 423 samples and 3,418 features, which mimics transcriptomic data. How would the benchmark results generalize to other high-dimensional omics data types such as genomic (e.g., GWAS) or epigenomic (e.g., DNA methylation) data, which typically have hundreds of thousands of features? In such ultra-high-dimensional settings, how feasible is it to compare the performance of different models computationally and statistically?

Our response: Thank you for the inquiry. As a primary goal with all of these omics data types is to find associations between patient time-to-event and certain quantities associated with variation on the genome, the results of this benchmark may be transferrable to these related data types. However, due to the drastic increase in data dimensionality in the case of this other data, we do not wish to make any suggestive comments that are outside the scope of our study.

2. When performing feature selection using filtering methods, did the authors examine QQ plots to evaluate whether test statistics followed the expected theoretical distributions? An excess of false-positive or false-negative findings during feature selection could substantially affect the downstream prognostic modeling performance.

Our response: Thank you for the inquiry. We have now briefly examined the distributions of test statistics for the filter methods in our studies. We have not examined QQ plots, but the overall distribution shapes appear to match what is expected (i.e. upward-skewed FDR-adjusted p-values and normal-like CARS scores). We agree that the false positivity and negativity rates of the selection methods may affect the downstream analysis. We did notice that in the real data analysis and our second setting of simulations, the FDR-controlling methods (BH and QV) had a greater proportion of small p-values in the univariate Cox regression than in our first setting of simulations, which may have resulted in more downstream false-positives for these methods. We suspect this to be due to greater multi-colinearity in the real data covariates than in our simulated data, but we do not want to make any suggestive comments outside of the scope of our study.

3.The analyses involve two major steps: feature selection and predictive modeling. Some approaches combine these steps, while others perform them separately. Can the authors clarify whether the observed performance differences may be attributed to this methodological distinction?

Our response: Thank you for the feedback, we have now briefly discussed how performing these steps simultaneously (embedded methods) versus sequentially (filter methods) may conflate with performance to the manuscript in the discussion section.
