# Supplementary material for: Benchmark of biomarker identification and prognostic modeling methods on diverse censored data
Source: PLoS One. 2026 Jun 16;21(6):e0351429. doi: 10.1371/journal.pone.0351429 (PMC13271465; doi:10.1371/journal.pone.0351429)
Supplement: Attachment — Submitted filename: Response_to_Reviewers_auresp_2.pdf [file pone.0351429.s007.pdf]

Response to the Reviewer 3

We would again like to thank you for reading our revised article and providing further valuable feedback. We have included your comments below in italics, followed by our responses. All changes and additions made to the submission following your recent suggestions are done in blue in the tracked changes version of the manuscript.

I have just two small suggestions that could further improve clarity for readers, if feasible. First, since you've settled on a priori tuning settings, a brief supplement note with typical wall-clock times and the rough number of hyperparameter evaluations per method would make the accuracy–compute trade-offs transparent without adding experiments.

Our response: We have now included Table 8 to the manuscript that provides details on the existing software implementations for tuning method hyperparameters, including function calls, the hyperparameters tuned by the functions, the search strategies, and the expected numbers of search steps. Since many of these details are inherent characteristic to the software-specific implementations, they are presented in "Discussion: Technical specifications." Typical wall-clock times of the examined methods as we deploy them are provided in Table 1, "Methods at a glance."

Second, the addition of time-dependent Brier was very helpful; would you consider adding a light calibration check at 1 and 3 years (intercept/slope with a predicted-vs-observed plot)...

Our response: We have now performed calibration checks at the 1, 3, and 5 year horizons in the form of predicted-by-observed plots in the real data analysis.

Using the fitted PH models for the parametric methods or the child node aggregate KM estimator for the tree-based methods, observations were grouped into five nearly equal sized bins depending upon their estimated survival probabilities at each horizon. Within each bin, a KM estimator was fit to the observations, and evaluated at the horizon to estimate the bin's observed survival probability. The bin's predicted survival probability at the horizon was the mean of the bin's individual predicted probabilities of survival from the original PH model or tree-based predictor. Every point on the plot was a bin's pair of 1) predicted survival probability and its 2) corresponding observed survival probability.

For every combination of examined method and clinical horizon, this process was done for all ten outer folds in the real data analysis. We have chosen to present an additional figure that

compiles all of these constructed calibration curves. It is in the supporting materials (S4) instead of the main text due to its large size.

...and if practical a single decision-curve at one horizon comparing the top models to treat-all/none? These quick visuals would nicely complement CI and Brier and help readers judge trustworthiness and clinical usefulness. If adding the decision curve is burdensome, the calibration plots alone would already round things out.

Our response: In our setting, we do not consider the effect of treatment on the outcome. More specifically, the dataset is observational, and the methods are compared with respect to their feature selection and predictive performance rather than treatment effect estimation. Therefore, for the sake of conciseness and focus, we have included only the calibration plots. We hope this is acceptable.
