# Supplementary material for: Benchmark of biomarker identification and prognostic modeling methods on diverse censored data
Source: PLoS One. 2026 Jun 16;21(6):e0351429. doi: 10.1371/journal.pone.0351429 (PMC13271465; doi:10.1371/journal.pone.0351429)
Supplement: Attachment — Submitted filename: response_to_reviewers.pdf [file pone.0351429.s008.pdf]

**Response to the Reviewer 3**

Thank you again for your time in reviewing our revisions and providing us with valuable feedback. Your comments have been listed below in italics, followed by our responses. Our edits and additions made to the main manuscript and appendix are done in blue in the tracked changes versions of the respective documents.

*The revision substantially strengthens the manuscript (added time-dependent Brier score, stability summaries, and calibration plots). One issue remains that I believe should be addressed before acceptance: in the TCGA analysis, the outcome-informed preliminary feature selection (CARS-based screening) appears to be performed once using the full cohort prior to nested CV. Because CARS uses outcome information (via correlations/weights), this can introduce subtle information leakage into cross-validated estimates. Please apply the screening step within each outer training fold (preferred), or provide a sensitivity analysis (e.g., within-fold screening for a subset of folds/methods) demonstrating that performance and top-feature conclusions are unchanged.*

**Our response:** Thank you for the insight regarding information leakage induced by performing the screening step prior to data splitting in the real data analysis. Although some information leakage may have occurred as a result of this decision, we have some concerns, primarily that performing the screening step per-fold would result in the analyzed methods performing feature selection from different feature sets each fold, which may confound their feature selection stability results and be less interpretable by readers. We have instead appended a brief sensitivity analysis of the preliminary feature selection method to the supporting information (S1 Appendix) that examines how stable the screening step is when applied per-fold, titled "Sensitivity analysis of PFS." We argue that the stability of the screening step suggests that the per-fold performance of the analyzed methods should vary little from what is presented in the article, so that the relative performances should remain unchanged. We hope that this will suffice.

Also please note that to further address concerns regarding information leakage, the real data analysis has been slightly modified so that feature standardization to mean 0 variance 1 occurred after performing the data split, and the test set observations were also shifted/scaled by the same values. Very slight performance change occurred in the results, but the relative rankings in the analysis remained unchanged along with our conclusions.

*As a small enhancement, adding a numeric calibration summary (e.g., intercept/slope or a calibration error metric at 1 and 3 years) alongside the existing calibration plots would improve interpretability. With these modest additions, the paper would be technically sound and a useful benchmark resource.*

**Our response:** Thank you for the further recommendations. To maintain our submission's conciseness, we have decided to report the LS intercept/slope corresponding to each of the calibration curves within the S4 Figure, with corresponding drawn regression lines.
