# Supplementary material for: Benchmark of biomarker identification and prognostic modeling methods on diverse censored data
Source: PLoS One. 2026 Jun 16;21(6):e0351429. doi: 10.1371/journal.pone.0351429 (PMC13271465; doi:10.1371/journal.pone.0351429)
Supplement: Peer review history

# Peer review: Decision Letter 0

**DOI:** 10.1371/journal.pone.0351429.r001

**Authors:** Guanghui Liu

12 Oct 2025

PONE-D-25-50851

Benchmark of biomarker identification and prognostic modeling methods on diverse censored data

PLOS ONE

Dear Dr. Fletcher,

Thank you for submitting your manuscript to PLOS ONE. After careful consideration, we feel that it has merit but does not fully meet PLOS ONE’s publication criteria as it currently stands. Therefore, we invite you to submit a revised version of the manuscript that addresses the points raised during the review process.

Please submit your revised manuscript by Nov 26 2025 11:59PM. If you will need more time than this to complete your revisions, please reply to this message or contact the journal office at plosone@plos.org. When you're ready to submit your revision, log on to https://www.editorialmanager.com/pone/ and select the 'Submissions Needing Revision' folder to locate your manuscript file.

Please include the following items when submitting your revised manuscript:

- A rebuttal letter that responds to each point raised by the academic editor and reviewer(s). You should upload this letter as a separate file labeled 'Response to Reviewers'.
- A marked-up copy of your manuscript that highlights changes made to the original version. You should upload this as a separate file labeled 'Revised Manuscript with Track Changes'.
- An unmarked version of your revised paper without tracked changes. You should upload this as a separate file labeled 'Manuscript'.

If you would like to make changes to your financial disclosure, please include your updated statement in your cover letter. Guidelines for resubmitting your figure files are available below the reviewer comments at the end of this letter.

If applicable, we recommend that you deposit your laboratory protocols in protocols.io to enhance the reproducibility of your results. Protocols.io assigns your protocol its own identifier (DOI) so that it can be cited independently in the future. For instructions see: https://journals.plos.org/plosone/s/submission-guidelines#loc-laboratory-protocols. Additionally, PLOS ONE offers an option for publishing peer-reviewed Lab Protocol articles, which describe protocols hosted on protocols.io. Read more information on sharing protocols at https://plos.org/protocols?utm_medium=editorial-email&utm_source=authorletters&utm_campaign=protocols.

We look forward to receiving your revised manuscript.

Kind regards,

Guanghui Liu

Academic Editor

PLOS ONE

Journal Requirements:

When submitting your revision, we need you to address these additional requirements.

1. Please ensure that your manuscript meets PLOS ONE's style requirements, including those for file naming. The PLOS ONE style templates can be found at https://journals.plos.org/plosone/s/file?id=wjVg/PLOSOne_formatting_sample_main_body.pdf and https://journals.plos.org/plosone/s/file?id=ba62/PLOSOne_formatting_sample_title_authors_affiliations.pdf

2. Thank you for stating the following financial disclosure:

This research was partially funded by the Texas A&M University College of Arts and Sciences 2025 Seed Grant.

Please state what role the funders took in the study. If the funders had no role, please state: "The funders had no role in study design, data collection and analysis, decision to publish, or preparation of the manuscript."

If this statement is not correct you must amend it as needed.

Please include this amended Role of Funder statement in your cover letter; we will change the online submission form on your behalf.

3. If the reviewer comments include a recommendation to cite specific previously published works, please review and evaluate these publications to determine whether they are relevant and should be cited. There is no requirement to cite these works unless the editor has indicated otherwise.

[Note: HTML markup is below. Please do not edit.]

Reviewers' comments:

Reviewer's Responses to Questions

**Comments to the Author**

1. Is the manuscript technically sound, and do the data support the conclusions?

The manuscript must describe a technically sound piece of scientific research with data that supports the conclusions. Experiments must have been conducted rigorously, with appropriate controls, replication, and sample sizes. The conclusions must be drawn appropriately based on the data presented.

Reviewer #1: Yes

Reviewer #2: Yes

Reviewer #3: Partly

Reviewer #4: Partly

**********

2. Has the statistical analysis been performed appropriately and rigorously?

Reviewer #1: Yes

Reviewer #2: Yes

Reviewer #3: No

Reviewer #4: Yes

**********

3. Have the authors made all data underlying the findings in their manuscript fully available?

The PLOS Data policy requires authors to make all data underlying the findings described in their manuscript fully available without restriction, with rare exception (please refer to the Data Availability Statement in the manuscript PDF file). The data should be provided as part of the manuscript or its supporting information, or deposited to a public repository. For example, in addition to summary statistics, the data points behind means, medians and variance measures should be available. If there are restrictions on publicly sharing data—e.g. participant privacy or use of data from a third party—those must be specified.

Reviewer #1: Yes

Reviewer #2: Yes

Reviewer #3: No

Reviewer #4: Yes

**********

4. Is the manuscript presented in an intelligible fashion and written in standard English?

PLOS ONE does not copyedit accepted manuscripts, so the language in submitted articles must be clear, correct, and unambiguous. Any typographical or grammatical errors should be corrected at revision, so please note any specific errors here.

Reviewer #1: Yes

Reviewer #2: Yes

Reviewer #3: No

Reviewer #4: Yes

**********

5. Review Comments to the Author

Please use the space provided to explain your answers to the questions above. You may also include additional comments for the author, including concerns about dual publication, research ethics, or publication ethics. (Please upload your review as an attachment if it exceeds 20,000 characters)

Reviewer #1: This manuscript investigates the comparative performance of methods for feature selection and survival prediction in high-dimensional right-censored survival data, using two simulation settings and a TCGA bladder cancer cohort.

Comments:

1. Sparsity levels. The paper states sparsity levels of 2%, 5%, and 10%, but on p.12 (Predictive performances), it refers to 1% and 5%.

2. Suggestion for Figures 7–9. The results are primarily presented with boxplots, which show relative ordering but make it hard for readers to see the size of the differences. Adding a compact summary table with the median CI and median RMSE for each method would make the improvements clearer.

Reviewer #2: The study examined several existing analysis methods to compare variable selection and survival prediction on both synthetic and TCGA dataset. The authors then manipulated features in generating synthetic datasets and evaluated the performance of each method using various criteria. Based on their results, the authors recommended ALASSO and CoxBoost as the most effective, among other recommendations. The overall significance and quality of the study can be further improved by addressing the following:

1. Line 271: Is there a particular reason the bladder cancer cohort is selected among other disease types and datasets available in the TCGA?

2. The authors reported DLEU1 deletion was selected by all methods except CARS and MSR. Was this target found or validated previously through experimental methods? Is it logically sound to assume that, with such rigorous methodology, well known drivers with mRNA level differences in various cancers should be discovered, and may serve as positive controls to the methods used in the study? If so, such observations can add credibility to the results and methods of the study.

Reviewer #3: Major comments

The paper should explicitly document where each data operation happens relative to cross-validation. Any feature screening, pilot fits for adaptive penalties, or hyperparameter tuning must be repeated strictly within training folds for every split. A concise data-flow diagram per fold would remove ambiguity. If nested cross-validation was not used for hyperparameter selection, please switch to it or justify the choice and quantify any optimistic bias.

Tuning budgets, search spaces, and early-stopping criteria appear uneven across algorithms. Key hyperparameters are not tuned under a common budget or search design across methods (e.g., broad λ paths for penalized Cox contrasted with largely default RSF settings), and there’s no nested, evaluation-count–matched inner loop; as a result, observed performance may conflate model quality with unequal search effort.

For the TCGA analysis, add time-dependent Brier score (or integrated Brier), and/or calibration assessments at clinically relevant horizons (e.g., 1- and 3-year). Make it explicit that RMSE on log-time is simulation-only since true event times are not observed with censoring in real data.

In the absence of ground truth for TCGA, please report selection stability. Bootstrapped selection frequencies, overlap heatmaps, and simple stability-selection summaries will help readers judge robustness. If conclusions about specific genes are highlighted, include their selection frequency and effect direction across folds.

Because the preliminary screening step materially shapes downstream results, quantify its impact. In simulations, show the rate at which true signals are removed by screening under varying signal-to-noise; in TCGA, present at least one robustness check where embedded methods are run with and without preliminary screening.

Ensure the benchmark isn’t biased toward L1-only Cox. So at least add Elastic-Net Cox (α∈{0.2, 0.5, 0.8}; tune λ with inner CV). This directly addresses correlated features—a common reality in omics—and materially affects conclusions about “best” pipelines.

Results on a single TCGA cohort limit generality. If feasible, add a second cancer type with similar preprocessing to show whether method rankings persist. If not, clearly caveat that BLCA is illustrative and avoid claims of general superiority.

Provide fixed seeds, fold assignments, and a repository snapshot (commit hash or DOI) with scripts to regenerate figures and tables. Export per-fold predictions and outcomes so third parties can independently recompute CI, Brier, and calibration. This materially increases the paper’s value as a community benchmark.

Minor comments

Standardize “censoring rate” (not “censorship”), correct typos, and ensure consistent naming of methods and packages.

Clarify which q-value implementation is used and specify π₀ estimation; if a nonstandard helper is employed, justify or switch to the canonical qvalue package for clarity.

For RSF, compare OOB and held-out metrics to rule out tuning mismatch. If you introduce any heuristic thresholding (e.g., MSR “elbow”), add a brief sensitivity analysis to show conclusions are not fragile to that choice.

Finally, add a short “methods-at-a-glance” table summarizing each pipeline (what is tuned, what is penalized, what the output is, and typical runtime) to improve usability for non-experts.

Reviewer #4: In this study, the authors compared various methods for identifying genomic biomarkers and developing prognostic models for time-to-event (survival) data. Genomic data are typically high-dimensional, correlated, and sparse, which makes feature selection (i.e., identifying informative biomarkers) challenging. Building upon existing methods that address these issues, the authors conducted a large-scale benchmark analysis using TCGA data to evaluate model performance across diverse right-censored survival datasets. By assessing multiple metrics—including Concordance Index, F1-Score, False Discovery Rate, Root Mean Square Error, and computation time—they found that CoxBoost performed well across all metrics, while LASSO and Adaptive LASSO achieved strong performance in terms of Concordance Index and F1-Score.

The study is well-designed, and the logical structure is sound. However, I have several concerns:

1.The synthetic dataset includes 423 samples and 3,418 features, which mimics transcriptomic data. How would the benchmark results generalize to other high-dimensional omics data types such as genomic (e.g., GWAS) or epigenomic (e.g., DNA methylation) data, which typically have hundreds of thousands of features? In such ultra-high-dimensional settings, how feasible is it to compare the performance of different models computationally and statistically?

2.When performing feature selection using filtering methods, did the authors examine QQ plots to evaluate whether test statistics followed the expected theoretical distributions? An excess of false-positive or false-negative findings during feature selection could substantially affect the downstream prognostic modeling performance.

3.The analyses involve two major steps: feature selection and predictive modeling. Some approaches combine these steps, while others perform them separately. Can the authors clarify whether the observed performance differences may be attributed to this methodological distinction?

**********

6. PLOS authors have the option to publish the peer review history of their article (what does this mean?). If published, this will include your full peer review and any attached files.

If you choose “no”, your identity will remain anonymous but your review may still be made public.

**Do you want your identity to be public for this peer review?**   For information about this choice, including consent withdrawal, please see our Privacy Policy.

Reviewer #1: No

Reviewer #2: No

Reviewer #3: No

Reviewer #4: No

**********

[NOTE: If reviewer comments were submitted as an attachment file, they will be attached to this email and accessible via the submission site. Please log into your account, locate the manuscript record, and check for the action link "View Attachments". If this link does not appear, there are no attachment files.]

While revising your submission, please upload your figure files to the Preflight Analysis and Conversion Engine (PACE) digital diagnostic tool, https://pacev2.apexcovantage.com/. PACE helps ensure that figures meet PLOS requirements. To use PACE, you must first register as a user. Registration is free. Then, login and navigate to the UPLOAD tab, where you will find detailed instructions on how to use the tool. If you encounter any issues or have any questions when using PACE, please email PLOS at figures@plos.org. Please note that Supporting Information files do not need this step.

# Author comment: Author response to Decision Letter 1

**DOI:** 10.1371/journal.pone.0351429.r002

3 Dec 2025

Thank you for your insightful comments and the opportunity to revise the manuscript. The point-by-point response to your comments is provided in the submission files, within the file named Response To Reviewers.pdf

Attachment Submitted filename: Response to Reviewers.pdf

# Peer review: Decision Letter 1

**DOI:** 10.1371/journal.pone.0351429.r003

**Authors:** Guanghui Liu

13 Jan 2026

PONE-D-25-50851R1

Benchmark of biomarker identification and prognostic modeling methods on diverse censored data

PLOS One

Dear Dr. Fletcher,

Thank you for submitting your manuscript to PLOS ONE. After careful consideration, we feel that it has merit but does not fully meet PLOS ONE’s publication criteria as it currently stands. Therefore, we invite you to submit a revised version of the manuscript that addresses the points raised during the review process.

Please submit your revised manuscript by Feb 27 2026 11:59PM. If you will need more time than this to complete your revisions, please reply to this message or contact the journal office at plosone@plos.org. When you're ready to submit your revision, log on to https://www.editorialmanager.com/pone/ and select the 'Submissions Needing Revision' folder to locate your manuscript file.

Please include the following items when submitting your revised manuscript:

- A letter that responds to each point raised by the academic editor and reviewer(s). You should upload this letter as a separate file labeled 'Response to Reviewers'.
- A marked-up copy of your manuscript that highlights changes made to the original version. You should upload this as a separate file labeled 'Revised Manuscript with Track Changes'.
- An unmarked version of your revised paper without tracked changes. You should upload this as a separate file labeled 'Manuscript'.

-->If you would like to make changes to your financial disclosure, please include your updated statement in your cover letter. Guidelines for resubmitting your figure files are available below the reviewer comments at the end of this letter.

If applicable, we recommend that you deposit your laboratory protocols in protocols.io to enhance the reproducibility of your results. Protocols.io assigns your protocol its own identifier (DOI) so that it can be cited independently in the future. For instructions see: https://journals.plos.org/plosone/s/submission-guidelines#loc-laboratory-protocols. Additionally, PLOS ONE offers an option for publishing peer-reviewed Lab Protocol articles, which describe protocols hosted on protocols.io. Read more information on sharing protocols at https://plos.org/protocols?utm_medium=editorial-email&utm_source=authorletters&utm_campaign=protocols.

We look forward to receiving your revised manuscript.

Kind regards,

Guanghui Liu

Academic Editor

PLOS One

**Journal Requirements:**

1. If the reviewer comments include a recommendation to cite specific previously published works, please review and evaluate these publications to determine whether they are relevant and should be cited. There is no requirement to cite these works unless the editor has indicated otherwise.

2. Please review your reference list to ensure that it is complete and correct. If you have cited papers that have been retracted, please include the rationale for doing so in the manuscript text, or remove these references and replace them with relevant current references. Any changes to the reference list should be mentioned in the rebuttal letter that accompanies your revised manuscript. If you need to cite a retracted article, indicate the article’s retracted status in the References list and also include a citation and full reference for the retraction notice.

[Note: HTML markup is below. Please do not edit.]

Reviewers' comments:

Reviewer's Responses to Questions

**Comments to the Author**

1. If the authors have adequately addressed your comments raised in a previous round of review and you feel that this manuscript is now acceptable for publication, you may indicate that here to bypass the “Comments to the Author” section, enter your conflict of interest statement in the “Confidential to Editor” section, and submit your "Accept" recommendation.

Reviewer #1: All comments have been addressed

Reviewer #3: (No Response)

Reviewer #4: All comments have been addressed

**********

2. Is the manuscript technically sound, and do the data support the conclusions?

The manuscript must describe a technically sound piece of scientific research with data that supports the conclusions. Experiments must have been conducted rigorously, with appropriate controls, replication, and sample sizes. The conclusions must be drawn appropriately based on the data presented.

Reviewer #1: Yes

Reviewer #3: Yes

Reviewer #4: Yes

**********

3. Has the statistical analysis been performed appropriately and rigorously?

Reviewer #1: Yes

Reviewer #3: Yes

Reviewer #4: Yes

**********

4. Have the authors made all data underlying the findings in their manuscript fully available?

The PLOS Data policy requires authors to make all data underlying the findings described in their manuscript fully available without restriction, with rare exception (please refer to the Data Availability Statement in the manuscript PDF file). The data should be provided as part of the manuscript or its supporting information, or deposited to a public repository. For example, in addition to summary statistics, the data points behind means, medians and variance measures should be available. If there are restrictions on publicly sharing data—e.g. participant privacy or use of data from a third party—those must be specified.

Reviewer #1: Yes

Reviewer #3: Yes

Reviewer #4: Yes

**********

5. Is the manuscript presented in an intelligible fashion and written in standard English?

PLOS ONE does not copyedit accepted manuscripts, so the language in submitted articles must be clear, correct, and unambiguous. Any typographical or grammatical errors should be corrected at revision, so please note any specific errors here.

Reviewer #1: Yes

Reviewer #3: Yes

Reviewer #4: Yes

**********

6. Review Comments to the Author

Please use the space provided to explain your answers to the questions above. You may also include additional comments for the author, including concerns about dual publication, research ethics, or publication ethics. (Please upload your review as an attachment if it exceeds 20,000 characters)

Reviewer #1: (No Response)

Reviewer #3: Thanks for the thoughtful revisions. I have just two small suggestions that could further improve clarity for readers, if feasible. First, since you’ve settled on a priori tuning settings, a brief supplement note with typical wall-clock times and the rough number of hyperparameter evaluations per method would make the accuracy–compute trade-offs transparent without adding experiments. Second, the addition of time-dependent Brier was very helpful; would you consider adding a light calibration check at 1 and 3 years (intercept/slope with a predicted-vs-observed plot), and if practical a single decision-curve at one horizon comparing the top models to treat-all/none? These quick visuals would nicely complement CI and Brier and help readers judge trustworthiness and clinical usefulness. If adding the decision curve is burdensome, the calibration plots alone would already round things out.

Reviewer #4: Thank you to the authors for their careful responses to my previous comments. The authors have adequately addressed all of my concerns. This manuscript compares the performance of feature-selection and survival-prediction methods for high-dimensional right-censored survival data using two simulation settings and a TCGA bladder cancer cohort. To my knowledge, few published studies have examined this topic in such detail. The manuscript is well structured, and the results are presented clearly.

**********

7. PLOS authors have the option to publish the peer review history of their article (what does this mean?). If published, this will include your full peer review and any attached files.

If you choose “no”, your identity will remain anonymous but your review may still be made public.

**Do you want your identity to be public for this peer review?**   For information about this choice, including consent withdrawal, please see our Privacy Policy.

Reviewer #1: No

Reviewer #3: No

Reviewer #4: No

**********

[NOTE: If reviewer comments were submitted as an attachment file, they will be attached to this email and accessible via the submission site. Please log into your account, locate the manuscript record, and check for the action link "View Attachments". If this link does not appear, there are no attachment files.]

To ensure your figures meet our technical requirements, please review our figure guidelines: https://journals.plos.org/plosone/s/figures

You may also use PLOS’s free figure tool, NAAS, to help you prepare publication quality figures: https://journals.plos.org/plosone/s/figures#loc-tools-for-figure-preparation.

NAAS will assess whether your figures meet our technical requirements by comparing each figure against our figure specifications.

# Author comment: Author response to Decision Letter 2

**DOI:** 10.1371/journal.pone.0351429.r004

29 Jan 2026

Thank you again for the opportunity to revise our manuscript. Our point-by-point response to your concerns can be found in the submission file titled "Response to Reviewers.pdf."

Attachment Submitted filename: Response_to_Reviewers_auresp_2.pdf

# Peer review: Decision Letter 2

**DOI:** 10.1371/journal.pone.0351429.r005

**Authors:** Guanghui Liu

13 Mar 2026

PONE-D-25-50851R2

Benchmark of biomarker identification and prognostic modeling methods on diverse censored data

PLOS One

Dear Dr. Fletcher,

Thank you for submitting your manuscript to PLOS ONE. After careful consideration, we feel that it has merit but does not fully meet PLOS ONE’s publication criteria as it currently stands. Therefore, we invite you to submit a revised version of the manuscript that addresses the points raised during the review process.

Please submit your revised manuscript by Apr 27 2026 11:59PM. If you will need more time than this to complete your revisions, please reply to this message or contact the journal office at plosone@plos.org. When you're ready to submit your revision, log on to https://www.editorialmanager.com/pone/ and select the 'Submissions Needing Revision' folder to locate your manuscript file.

Please include the following items when submitting your revised manuscript:

- A letter that responds to each point raised by the academic editor and reviewer(s). You should upload this letter as a separate file labeled 'Response to Reviewers'.
- A marked-up copy of your manuscript that highlights changes made to the original version. You should upload this as a separate file labeled 'Revised Manuscript with Track Changes'.
- An unmarked version of your revised paper without tracked changes. You should upload this as a separate file labeled 'Manuscript'.

-->If you would like to make changes to your financial disclosure, please include your updated statement in your cover letter. Guidelines for resubmitting your figure files are available below the reviewer comments at the end of this letter.

If applicable, we recommend that you deposit your laboratory protocols in protocols.io to enhance the reproducibility of your results. Protocols.io assigns your protocol its own identifier (DOI) so that it can be cited independently in the future. For instructions see: https://journals.plos.org/plosone/s/submission-guidelines#loc-laboratory-protocols. Additionally, PLOS ONE offers an option for publishing peer-reviewed Lab Protocol articles, which describe protocols hosted on protocols.io. Read more information on sharing protocols at https://plos.org/protocols?utm_medium=editorial-email&utm_source=authorletters&utm_campaign=protocols.

We look forward to receiving your revised manuscript.

Kind regards,

Guanghui Liu

Academic Editor

PLOS One

Journal Requirements:

If the reviewer comments include a recommendation to cite specific previously published works, please review and evaluate these publications to determine whether they are relevant and should be cited. There is no requirement to cite these works unless the editor has indicated otherwise.

Please review your reference list to ensure that it is complete and correct. If you have cited papers that have been retracted, please include the rationale for doing so in the manuscript text, or remove these references and replace them with relevant current references. Any changes to the reference list should be mentioned in the rebuttal letter that accompanies your revised manuscript. If you need to cite a retracted article, indicate the article’s retracted status in the References list and also include a citation and full reference for the retraction notice.

[Note: HTML markup is below. Please do not edit.]

Reviewers' comments:

Reviewer's Responses to Questions

**Comments to the Author**

1. If the authors have adequately addressed your comments raised in a previous round of review and you feel that this manuscript is now acceptable for publication, you may indicate that here to bypass the “Comments to the Author” section, enter your conflict of interest statement in the “Confidential to Editor” section, and submit your "Accept" recommendation.

Reviewer #1: All comments have been addressed

Reviewer #3: (No Response)

Reviewer #4: All comments have been addressed

**********

2. Is the manuscript technically sound, and do the data support the conclusions?

The manuscript must describe a technically sound piece of scientific research with data that supports the conclusions. Experiments must have been conducted rigorously, with appropriate controls, replication, and sample sizes. The conclusions must be drawn appropriately based on the data presented.

Reviewer #1: (No Response)

Reviewer #3: Partly

Reviewer #4: Yes

**********

3. Has the statistical analysis been performed appropriately and rigorously?

Reviewer #1: (No Response)

Reviewer #3: Yes

Reviewer #4: Yes

**********

4. Have the authors made all data underlying the findings in their manuscript fully available?

The PLOS Data policy requires authors to make all data underlying the findings described in their manuscript fully available without restriction, with rare exception (please refer to the Data Availability Statement in the manuscript PDF file). The data should be provided as part of the manuscript or its supporting information, or deposited to a public repository. For example, in addition to summary statistics, the data points behind means, medians and variance measures should be available. If there are restrictions on publicly sharing data—e.g. participant privacy or use of data from a third party—those must be specified.

Reviewer #1: (No Response)

Reviewer #3: Yes

Reviewer #4: Yes

**********

5. Is the manuscript presented in an intelligible fashion and written in standard English?

PLOS ONE does not copyedit accepted manuscripts, so the language in submitted articles must be clear, correct, and unambiguous. Any typographical or grammatical errors should be corrected at revision, so please note any specific errors here.

Reviewer #1: (No Response)

Reviewer #3: Yes

Reviewer #4: Yes

**********

6. Review Comments to the Author

Please use the space provided to explain your answers to the questions above. You may also include additional comments for the author, including concerns about dual publication, research ethics, or publication ethics. (Please upload your review as an attachment if it exceeds 20,000 characters)

Reviewer #1: (No Response)

Reviewer #3: The revision substantially strengthens the manuscript (added time-dependent Brier score, stability summaries, and calibration plots). One issue remains that I believe should be addressed before acceptance: in the TCGA analysis, the outcome-informed preliminary feature selection (CARS-based screening) appears to be performed once using the full cohort prior to nested CV. Because CARS uses outcome information (via correlations/weights), this can introduce subtle information leakage into cross-validated estimates. Please apply the screening step within each outer training fold (preferred), or provide a sensitivity analysis (e.g., within-fold screening for a subset of folds/methods) demonstrating that performance and top-feature conclusions are unchanged. As a small enhancement, adding a numeric calibration summary (e.g., intercept/slope or a calibration error metric at 1 and 3 years) alongside the existing calibration plots would improve interpretability. With these modest additions, the paper would be technically sound and a useful benchmark resource.

Reviewer #4: The authors have carefully addressed all of my previous comments and provided clear and satisfactory clarifications regarding the bioinformatic preprocessing steps, the selection of mitophagy-related genes, and the modeling workflow.

**********

7. PLOS authors have the option to publish the peer review history of their article (what does this mean?). If published, this will include your full peer review and any attached files.

If you choose “no”, your identity will remain anonymous but your review may still be made public.

**Do you want your identity to be public for this peer review?**   For information about this choice, including consent withdrawal, please see our Privacy Policy.

Reviewer #1: No

Reviewer #3: No

Reviewer #4: No

**********

[NOTE: If reviewer comments were submitted as an attachment file, they will be attached to this email and accessible via the submission site. Please log into your account, locate the manuscript record, and check for the action link "View Attachments". If this link does not appear, there are no attachment files.]

To ensure your figures meet our technical requirements, please review our figure guidelines: https://journals.plos.org/plosone/s/figures

You may also use PLOS’s free figure tool, NAAS, to help you prepare publication quality figures: https://journals.plos.org/plosone/s/figures#loc-tools-for-figure-preparation.

NAAS will assess whether your figures meet our technical requirements by comparing each figure against our figure specifications.

# Author comment: Author response to Decision Letter 3

**DOI:** 10.1371/journal.pone.0351429.r006

30 Mar 2026

Thank you for the continued correspondence and providing us with helpful comments and recommendations. Our full point-by-point response is included in the file response_to_reviewers.pdf with our submission.

Attachment Submitted filename: response_to_reviewers.pdf

# Peer review: Decision Letter 3

**DOI:** 10.1371/journal.pone.0351429.r007

**Authors:** Michael Brimacombe

28 May 2026

Benchmark of biomarker identification and prognostic modeling methods on diverse censored data

PONE-D-25-50851R3

Dear Wesley Fletcher,

We’re pleased to inform you that your manuscript has been judged scientifically suitable for publication and will be formally accepted for publication once it meets all outstanding technical requirements.

Within one week, you’ll receive an e-mail detailing the required amendments. When these have been addressed, you’ll receive a formal acceptance letter and your manuscript will be scheduled for publication.

An invoice will be generated when your article is formally accepted. Please note, if your institution has a publishing partnership with PLOS and your article meets the relevant criteria, all or part of your publication costs will be covered. Please make sure your user information is up-to-date by logging into Editorial Manager at Editorial Manager® and clicking the ‘Update My Information' link at the top of the page. For questions related to billing, please contact billing support.

If your institution or institutions have a press office, please notify them about your upcoming paper to help maximize its impact. If they’ll be preparing press materials, please inform our press team as soon as possible -- no later than 48 hours after receiving the formal acceptance. Your manuscript will remain under strict press embargo until 2 pm Eastern Time on the date of publication. For more information, please contact onepress@plos.org.

Kind regards,

Michael Brimacombe

Academic Editor

PLOS One

Additional Editor Comments (optional):

The paper is a very detailed comparison of various methods related to the statistical modeling of survival data in the context of various data structures. One additional comment from Reviewer 3: "One remaining methodological nuance is that the outcome-informed preliminary screening for the TCGA analysis is performed on the full cohort rather than within each outer training fold." This comment can be disregarded as the basic recruitment inclusion/exclusion is typically applied to the overall cohort. Otherwise, the reviewer comments have been addressed.

Reviewers' comments:

Reviewer's Responses to Questions

**Comments to the Author**

1. If the authors have adequately addressed your comments raised in a previous round of review and you feel that this manuscript is now acceptable for publication, you may indicate that here to bypass the “Comments to the Author” section, enter your conflict of interest statement in the “Confidential to Editor” section, and submit your "Accept" recommendation.

Reviewer #1: All comments have been addressed

Reviewer #3: All comments have been addressed

Reviewer #4: All comments have been addressed

**********

2. Is the manuscript technically sound, and do the data support the conclusions?

The manuscript must describe a technically sound piece of scientific research with data that supports the conclusions. Experiments must have been conducted rigorously, with appropriate controls, replication, and sample sizes. The conclusions must be drawn appropriately based on the data presented.

Reviewer #1: (No Response)

Reviewer #3: Yes

Reviewer #4: Yes

**********

3. Has the statistical analysis been performed appropriately and rigorously?

Reviewer #1: (No Response)

Reviewer #3: Yes

Reviewer #4: Yes

**********

4. Have the authors made all data underlying the findings in their manuscript fully available?

The PLOS Data policy requires authors to make all data underlying the findings described in their manuscript fully available without restriction, with rare exception (please refer to the Data Availability Statement in the manuscript PDF file). The data should be provided as part of the manuscript or its supporting information, or deposited to a public repository. For example, in addition to summary statistics, the data points behind means, medians and variance measures should be available. If there are restrictions on publicly sharing data—e.g. participant privacy or use of data from a third party—those must be specified.

Reviewer #1: (No Response)

Reviewer #3: Yes

Reviewer #4: Yes

**********

5. Is the manuscript presented in an intelligible fashion and written in standard English?

PLOS ONE does not copyedit accepted manuscripts, so the language in submitted articles must be clear, correct, and unambiguous. Any typographical or grammatical errors should be corrected at revision, so please note any specific errors here.

Reviewer #1: (No Response)

Reviewer #3: Yes

Reviewer #4: Yes

**********

6. Review Comments to the Author

Please use the space provided to explain your answers to the questions above. You may also include additional comments for the author, including concerns about dual publication, research ethics, or publication ethics. (Please upload your review as an attachment if it exceeds 20,000 characters)

Reviewer #1: (No Response)

Reviewer #3: The authors have substantially strengthened the manuscript in response to reviewer feedback, including adding time-dependent Brier score and calibration checks, providing feature-selection stability summaries, and clarifying the benchmarking workflow. One remaining methodological nuance is that the outcome-informed preliminary screening for the TCGA analysis is performed on the full cohort rather than within each outer training fold; I recommend that this be clearly stated as a limitation (and/or highlighted where the TCGA results are discussed). Overall, the revised work is technically sound and will be a useful benchmark reference.

Reviewer #4: The authors have adequately addressed my previous comments and concerns. The manuscript has been substantially improved, and I believe it is now suitable for publication.

**********

7. PLOS authors have the option to publish the peer review history of their article (what does this mean?). If published, this will include your full peer review and any attached files.

If you choose “no”, your identity will remain anonymous but your review may still be made public.

**Do you want your identity to be public for this peer review?**   For information about this choice, including consent withdrawal, please see our Privacy Policy.

Reviewer #1: No

Reviewer #3: No

Reviewer #4: No

**********

# Editor report: Acceptance letter

**DOI:** 10.1371/journal.pone.0351429.r008

**Authors:** Michael Brimacombe

PONE-D-25-50851R3

PLOS One

Dear Dr. Fletcher,

I'm pleased to inform you that your manuscript has been deemed suitable for publication in PLOS One. Congratulations! Your manuscript is now being handed over to our production team.

At this stage, our production department will prepare your paper for publication. This includes ensuring the following:

* All references, tables, and figures are properly cited

* All relevant supporting information is included in the manuscript submission,

* There are no issues that prevent the paper from being properly typeset

You will receive further instructions from the production team, including instructions on how to review your proof when it is ready. Please keep in mind that we are working through a large volume of accepted articles, so please give us a few days to review your paper and let you know the next and final steps.

Lastly, if your institution or institutions have a press office, please let them know about your upcoming paper now to help maximize its impact. If they'll be preparing press materials, please inform our press team within the next 48 hours. Your manuscript will remain under strict press embargo until 2 pm Eastern Time on the date of publication. For more information, please contact onepress@plos.org.

You will receive an invoice from PLOS for your publication fee after your manuscript has reached the completed accept phase. If you receive an email requesting payment before acceptance or for any other service, this may be a phishing scheme. Learn how to identify phishing emails and protect your accounts at https://explore.plos.org/phishing.

If we can help with anything else, please email us at customercare@plos.org.

Thank you for submitting your work to PLOS ONE and supporting open access.

Kind regards,

PLOS ONE Editorial Office Staff

on behalf of

Dr. Michael Brimacombe

Academic Editor

PLOS One
